# Responding to Positive Emotions at Work – The Four Steps and Potential Benefits of a Validating Response to Coworkers’ Positive Experiences

**DOI:** 10.3389/fpsyg.2021.668160

**Published:** 2021-10-11

**Authors:** Miia A. Paakkanen, Frank Martela, Anne B. Pessi

**Affiliations:** ^1^Theology, University of Helsinki, Helsinki, Finland; ^2^Industrial Engineering and Management, Aalto University, Espoo, Finland

**Keywords:** validating response, positive empathy, positive emotions, capitalization, psychological safety, interpersonal relationships, organizations

## Abstract

In order to capitalize on positive emotions at work and build high-quality interpersonal relationships and psychological safety, it is important that coworkers respond to each other’s positive emotions in a constructive and validating way. However, despite the importance of symmetrical emotion regulation outcomes, organizational research has largely overlooked how an employee can positively respond to coworkers’ positive emotions. Existing research has concentrated almost exclusively on negative ways of responding, with a particular focus on envy. This article develops a theoretical model of employees’ positive responses to coworkers’ positive emotional experiences, introduced here as a validating response. We identify four steps – noticing, sensemaking, feeling, and acting – and the key mechanisms within each step that enable a responder to react in a validating way. We connect the validating response to important potential individual and organizational outcomes. These outcomes include improved relationship quality and trust, as well as increased positivity and well-being that can result in enhanced learning behavior and collaboration. This article also discusses the connection between a validating response and compassion. We identify them both as parallel affirmative processes that acknowledge a coworker’s emotions, with the former being a response to positive emotion while the latter is a response to negative emotion.

## Introduction

Laura turns to her colleague Jenny: *“I got a heart-warming text message from a client. She gave me such encouraging feedback for my work for them that, when I first read it, tears came into my eyes*.” Jenny keeps her face still and her mouth shut and shows no facial or bodily expressions, nor does she say anything to Laura. Afterward, Laura comments on Jenny’s lack of facial reaction: *“I felt like she was not interested at all in what I had to say or what I was feeling, and I started feeling like there was something very stupid in what I was sharing. I lost confidence in myself, and Jenny’s reaction hurt me. I wanted to stop speaking to her.”* If Jenny had responded by joining in with Laura’s celebration of the good news, this moment could have strengthened the mood of both participants and deepened their mutual bond. By ignoring Laura’s joy, Jenny undermined their relationship and this had a detrimental effect on Laura’s mood.

This story is drawn from an exercise completed at an emotional skills training session for managers, and it provides an example of a typical work situation. A person wants to share their joy or good news with others, and they seek a positive response in order to capitalize on their positive experience and reinforce their positive emotions (Langston, 1994; Gable et al., 2004; Hadley, 2014). Interpersonal research has shown that this capitalization can lead to many positive outcomes, for example, subjective well-being, lesser negative affect, greater trust, and better relationship quality (Reis et al., 2010; Peters et al., 2018). However, the research has also shown that these positive outcomes are dependent on the responder reacting in an active and validating way rather than demonstrating a passive and unconstructive response (Gable et al., 2004). The discloser requires a positive response that acknowledges, validates, and reinforces their positive emotion – referred to here as a *validating response* – to ensure the experience leads to positive emotion regulation outcomes instead of social distress and dampening of the original feeling (Hadley, 2014). Many employees choose not to share positive emotions with others as they anticipate an asymmetrical response from their coworkers who may express envy or resentment rather than share their joy (Hadley, 2014). Therefore, in a situation where the discloser is relying on others to react positively, they take an interpersonal risk when they share their positive emotions and good news (Edmondson, 1999).

The aim of the present article was to examine the process of responding in a validating way to the positive emotions expressed by others. A validating response is an important building block for interpersonal capitalization (Hadley, 2014), high-quality connections (HQCs) (Dutton, 2003), and psychological safety (Edmondson, 1999) – workplace qualities that improve creativity, innovation, learning, and individual and team performance (Porath et al., 2012; Edmondson and Lei, 2014; Madrid et al., 2014; Frazier et al., 2017).

At a fundamental level, social connections are a basic human need (Baumeister and Leary, 1995; Deci and Ryan, 2000) and social rejection has been linked to emotional pain that is similar to physical pain (Eisenberger et al., 2003). As an integral part of feeling and strengthening social connection, expressions of positive emotions at work can benefit the individual expressing them as well as the coworkers, the customers, and the entire organization (Fredrickson, 2001; Reis et al., 2010). However, negative and ignorant reactions from coworkers can prevent the positive health effects of positive emotions (Gable et al., 2004; Kashdan et al., 2013) and create a direct source of suffering. Relationship studies have shown that asymmetrical outcomes of expressing positive emotions lead to a decrease in both the amount of positive emotions experienced and the perceived quality of the relationship (e.g., Gable et al., 2004; Kashdan et al., 2013). Furthermore, distress caused by the lack of a positive response reduces collaboration by undermining trust between colleagues and impeding the other building blocks of positive workplace relationships (Carmeli et al., 2015). The absence of a positive response is an interpersonal risk, and even its anticipation can undermine psychological safety within teams and limit essential behaviors for learning and collaboration, such as sharing ideas, questions, and feedback (Edmondson, 1999). Currently, little is known about “how psychological safety unfolds and builds, or lessens, or even is destroyed” (Edmondson and Lei, 2014: 38). How a person responds to the positive emotions of others is a particular aspect of interpersonal dynamics that could play an important role in building or decreasing psychological safety.

To date, research has largely overlooked the ways in which an employee can respond in a positive way to a colleague’s joy. Although research, for example, within emotional intelligence (EI; Mayer and Salovey, 1997; Côté, 2014), has examined the general ability to perceive and understand others’ emotions, Ganegoda and Bordia (2019: 776) recently stated that “no organizational research to date has looked at such *positive* responses to coworkers’ positive experiences.” Instead, previous research has focused on negative ways of responding, with envy receiving particular attention (e.g., Duffy et al., 2012; Tai et al., 2012). In general, the study of organizational behavior now requires a more strengths-focused approach (Luthans, 2002). To counteract the negative focus, Ganegoda and Bordia (2019) propose that studies should examine positive empathy – the experience of happiness in response to a coworker’s positive experience – in the organizational context. Building on their important contribution, this paper expands the focus to include the whole process of responding in a validating way to a coworker’s positive emotional experience. We propose that this process involves multiple steps and mechanisms, and positive empathy is just one, albeit crucial, step. Research has identified compassion in organizations as an empathic concern that is part of the process of responding to the suffering of others (see Dutton et al., 2014). Positive empathy is analogous to empathic concern, and its study presents one key part of the unfolding of the validating process. However, the entire process of responding to positive emotions in a validating way also involves other steps.

Ganegoda and Bordia (2019) recently published work on positive empathy, and Hadley (2014) has researched the emotion regulation outcomes of sharing positive and negative work events. Overall, however, the perspective of how the responder can react to positive emotions in a constructive way has largely been overlooked in organizational behavior literature – and also in interpersonal research (see Peters et al., 2018). Although a symmetrical emotion regulation outcome to a coworker’s expressions of positive emotions is of significant importance, there is a lack of research covering the validating response from the perspective of the responder.

Therefore, the purpose of this paper was to provide a theoretical and conceptual elaboration of employees’ positive responses to coworkers’ positive emotional experiences, referred to in this article as the validating response. We first review existing research on interpersonal dynamics in the workplace that underscore the potential significance of improving the understanding of these responses. A model is then presented to facilitate the study of the interpersonal process of responding to the positive emotions of others at work. This model uses four steps – noticing, sensemaking, feeling, and acting –that are studied individually to identify the potential mechanisms that enable a validating respond to others’ positive emotions in order to bring about desired symmetrical outcomes. We also explore the potential outcomes of a validating response in the workplace and connect employees’ validating responses to enhanced learning behavior and collaboration, relationship quality and trust, and increased positive affect and well-being. The article concludes with a discussion of the theoretical contributions and practical implications of the current study. We also highlight important avenues for future research that could broaden the understanding of this important aspect of work-based interpersonal emotion regulation dynamics. By providing a theoretical account of the validating response to the positive emotions of others, this article lays the groundwork for future researchers. The development and testing of theories that explore the expression and sharing of positive emotional experiences and their attentive responses should be a focus for fostering positive interpersonal dynamics and individual and collective well-being in organizations (Quinn and Dutton, 2005; Porath et al., 2012).

### Positive Emotions and Interpersonal Capitalization

Research on organizational behavior has increasingly acknowledged how experiences at work are “saturated with emotions” (Ashforth and Humphrey, 1995: 97; Weiss and Cropanzano, 1996) and how affective processes carry important interpersonal functions that play a crucial role in how organizational life unfolds (Barsade, 2002; Brief and Weiss, 2002). For example, Amabile et al. (2005) have shown how positive affect in organizations works both as an antecedent and as a consequence of creative thought, establishing an affect-creativity cycle. Barbara Fredrickson’s broaden and build theory (Fredrickson, 1998, 2001, 2004; Fredrickson and Joiner, 2002) demonstrated that positive emotions expand the momentary scopes of attention and cognition and thereby widen the array of thoughts and actions that come to mind. Positive emotions further build people’s enduring psychological, intellectual, and social resources (Fredrickson, 2001, 2004). Employees and organizations that have a reserve of positive emotions can draw on them to increase resilience in times of crisis or stress (Fredrickson, 2004; Powley, 2009). Furthermore, research has shown that people who report greater positive affect in general are better at social interactions and have interactions that are of a higher quality (Berry and Hansen, 1996). Overall, studies suggest that there are multiple benefits for employees when they experience an increase in positive emotions, including improved work performance (Amabile and Kramer, 2007; Sekerka et al., 2011; Kiffin-Petersen et al., 2012).

People often discuss positive events with others in the hope of sustaining, prolonging, or amplifying the positive emotions related to those events (Rimé, 2007). However, the success of such retelling in order to gain additional advantage from the events – a process called capitalization (Langston, 1994) – is vulnerable to the response of the person to whom the news is being told (i.e., the responder) (Gable et al., 2004). The retelling will only lead to positive outcomes when the individual recounting their good news (i.e., the capitalizer) perceives the response of the responder as active and constructive (understanding, validating, and caring) as opposed to passive or unconstructive (not valuing, disinterested, jealous, or self-absorbed) (Gable et al., 2004). The consequences of an unconstructive response may not be restricted to unrealized positive outcomes as they can also cause suffering and a perceived decrease in the quality of the relationship between the capitalizer and the responder (Gable et al., 2004; Kashdan et al., 2013). The role of the responder is therefore critical as they enable the positive outcomes of capitalization. Despite their important role in preventing negative outcomes, only limited research has focused on the perspective of the responder and the methods for forming a constructive response. In the last decade, researchers have acknowledged the interpersonal nature of capitalization and begun to explore the benefits of capitalization for the responder (e.g., Reis et al., 2010; Monfort et al., 2014; Conoley et al., 2015). However, research on the potential mechanisms through which the responder can hinder or promote the capitalization is largely missing (Peters et al., 2018). The studies have also primarily focused on the sharing of positive events (e.g., Peters et al., 2018) and overlooked the more general sharing of positive emotions that a recent positive event may or may not have triggered. Hadley (2014) observed that the majority (78%) of emotional incidents reported in her study on human service workers included a coworker interaction. Despite the frequency of these interactions, only a small number of studies have examined capitalization and interpersonal regulation of emotions through social interactions at work. The act of sharing emotions, both positive and negative, is prevalent in the workplace, and it has critical affective and relational consequences. However, the realization of positive outcomes is dependent on whether such sharing is met with a constructive and validating response. Overlooking the perspective of the responder results in a significant loss of information about interpersonal dynamics at work (Maisel and Gable, 2009). How colleagues respond to the positive emotional experiences of others presents an important influence on interpersonal capitalization that potentially enhances positive emotions at work, improves creativity, and benefits the overall well-being and performance of employees.

### Psychological Safety

Another important research stream on positive interpersonal responding concerns psychological safety – “people’s perceptions of the consequences of taking interpersonal risks in a particular context such as a workplace” – which organizational research has recognized as an essential factor in understanding interpersonal dynamics, such as team learning, voice, and collaboration (Edmondson and Lei, 2014: 23). In her seminal work, Edmondson (1999) showed that psychological safety among employees correlates with team learning. Following the publication of this influential research, an increasing number of studies have linked high levels of psychological safety with team performance, learning behaviors, and other critically important organizational outcomes (Edmondson and Lei, 2014; Frazier et al., 2017; O’Donovan and McAuliffe, 2020).

Learning and working collaboratively are integral parts of organizational life, especially in the current knowledge economies that are increasingly dependent on teamwork. However, these environments are often more interpersonally difficult than anticipated due to the high levels of trust required between team members. Psychological safety does not emerge spontaneously and often requires intentional effort on the part of the team members and the team leaders (Edmondson, 2003). Accordingly, academics have called for more research to explore how psychological safety is both built and destroyed (Edmondson and Lei, 2014).

This article argues that the interpersonal process of responding to the positive emotional experiences of others is an important factor influencing psychological safety. William Kahn (1990: 694) suggested that psychological safety positively affects individuals’ willingness to “employ or express themselves physically, cognitively, and emotionally during role performance” and prevents a move to disengage or “withdraw and defend their personal selves.” An important part of this effect is whether an individual has the courage and willingness to express their emotions, also positive ones. Expressing one’s positive emotional experiences at work is affected by how others respond to such expressions (Hadley, 2014). For example, studies have shown that employees are less motivated to share positive emotional experiences than negative ones due to a fear of a dampening response from their colleagues (Hadley, 2014). A person’s positive response to their coworker’s positive emotional experience can present an important factor reducing such fear and instead help build psychological safety, expressed as an increased willingness to share positive emotions. Consequently, a validating response could help enhance learning behavior and performance through increased sense of psychological safety.

### High-Quality Connections

Interpersonal responding can be also approached through research on HQCs, which have been identified as a life-giving force in the relational fabric of organizational life (Dutton, 2003) as they positively affect collaboration, relationship quality, and trust among colleagues (e.g., Dutton and Heaphy, 2003; Stephens et al., 2011). HQCs are defined as short-term, dyadic interactions where both the subjective experience of the connected individuals and their relationships are experienced as positive (Stephens et al., 2011). These shorter-term connective moments within ongoing relationships or encounters have also been described as important for building the interpersonal relationships at work (Dutton and Heaphy, 2003). A focus on short-term connections that can positively affect employee well-being and interpersonal dynamics, even between unfamiliar colleagues, may present a valuable resource for current fast-paced collaboration-dependent work life.

Studies that have explored how HQCs are formed and strengthened are limited, and further research is required to fully understand the potential mechanisms that enable the development of HQCs (Stephens et al., 2011). To date, research has suggested the involvement of various different emotional, cognitive, and behavioral mechanisms, such as empathy, perspective taking, and respectful engagement; however, the focus should now shift to identifying how these mechanisms might be interrelated (Stephens et al., 2011). This article asserts that the interpersonal process of responding to the positive emotional experiences of others presents an important pathway to build HQCs. By exploring the potential mechanisms of such responses, we can also extend the understanding of the potential mechanisms that enable HQCs. This research will show how employees can shape interactions at work more positively and strengthen collaboration, relationship quality, and trust.

### Compassion at Work

Compassion as a form of empathic concern provides an important parallel for present research. In organizational research, it has been defined as “an interpersonal process involving the noticing, feeling, sensemaking, and acting that alleviate the suffering of another person” (Dutton et al., 2014: 277). This process is initiated by a pain trigger and an expression of suffering by the sufferer. The compassionate actor then reacts to bring about positive outcomes for the sufferer, the responder, and the third parties witnessing the unfolding of the compassion process (Lilius et al., 2008; Dutton et al., 2014). Compassion thus focuses on the responder’s way of reacting to the emotional event of the discloser (Lilius et al., 2008; Dutton et al., 2014). The act of compassion represents a process that is parallel to the validating response examined in this article. The compassion process focuses on how to constructively respond to a discloser’s painful emotions, while the validating process focuses on how to constructively respond to a discloser’s positive emotions. The mechanisms of the two processes are likely to differ as the related aversive and appetitive processes operate independently (Cacioppo et al., 1997; Maisel and Gable, 2009; Hadley, 2014). However, the interpersonal process of responding to others’ suffering provides a useful framework that can be used in part to articulate the validating response. Researchers studying compassion argue that it is a process consisting of noticing, feeling, sensemaking, and acting, and these four steps are also required in our model of the validating response.

## The Interpersonal Process of Responding to the Positive Emotions of Others in a Validating Way

**Figure 1:**
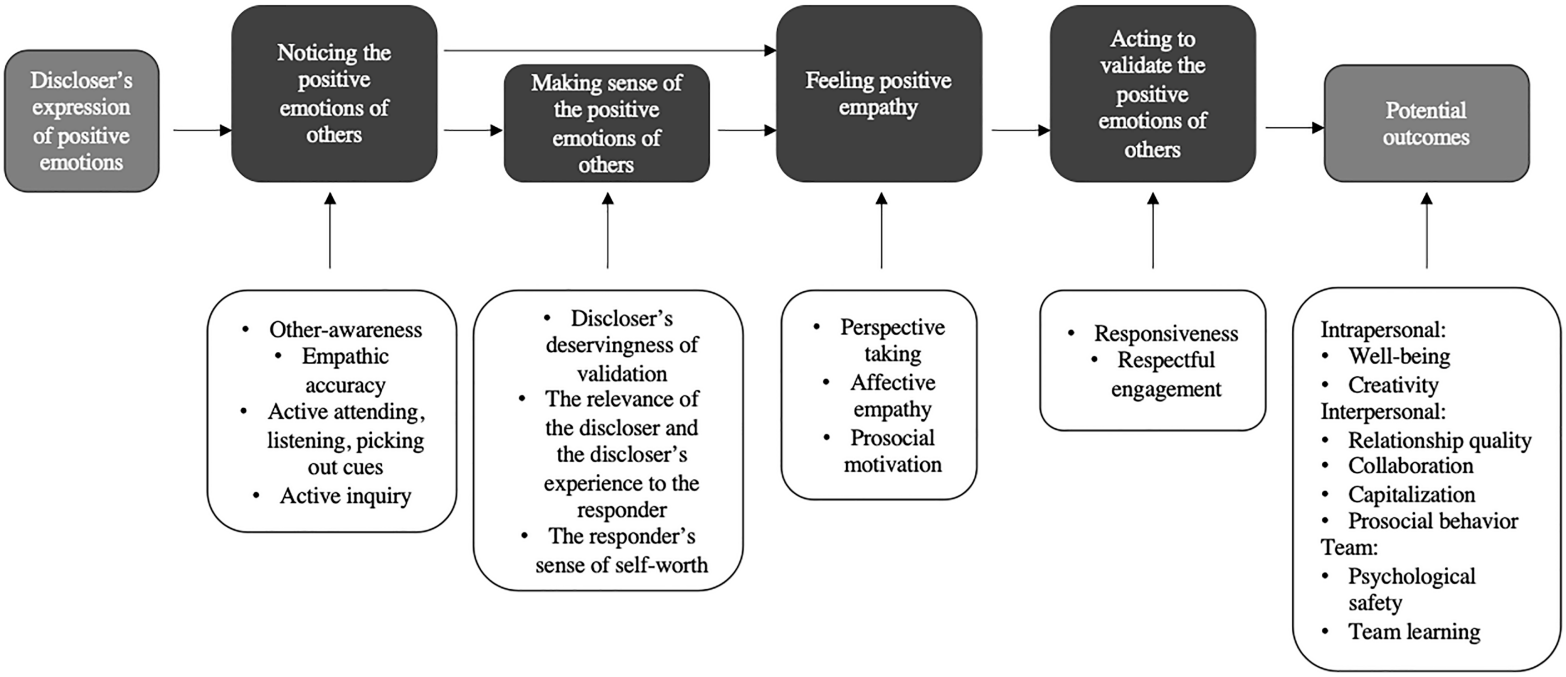
The process of responding to validate the positive emotions of coworkers (partially adapted from Dutton et al., 2014).

This study focuses on the responder and how they react to a discloser’s positive emotions in a validating way. We propose that the process involves the four steps of noticing, sensemaking, feeling, and acting. Within each of the four steps, we explore potential cognitive, emotional, and behavioral mechanisms that enable the validating process to occur between coworkers. As described by Stephens et al. (2011), cognitive mechanisms describe the conscious and unconscious thought processes that allow employees to respond in a validating way. Emotional mechanisms describe how emotions reveal a person’s thoughts and feelings and how they can be shared between colleagues to enable a validating response. Behavioral mechanisms, in turn, describe the various concrete ways of behaving and reacting to each other through which validation (or lack thereof) is expressed. Although the process is presented here as linear, unfolding in a sequential order (see Figure 1), in real-life situations, there can also be iterative back-and-forth movement that prepares the responder for action. Rather than provide an extensive account of every mechanism within each step, the aim of this study is to highlight only the key mechanisms.

### Noticing the Positive Emotions of Others

The validating response begins when the responder notices the other person’s positive emotions. The validating process will not initiate if the responder fails to perceive the discloser’s positive emotional experience. In a work environment, the process of noticing involves a responder who has become aware of a colleague’s emotional event by using their ongoing experience to observe relevant cues for conscious processing (Kanov et al., 2004; O’Donohoe and Turley, 2006). Within research on emotional intelligence, researchers have highlighted perceiving and expressing emotions as one key branch of EI (Mayer and Salovey, 1997; Côté, 2014). Noticing other’s positive emotion is dependent on one’s emotion recognition ability utilizing both non-verbal and verbal cues (Rubin et al., 2005; Côté, 2014) and more generally on other-awareness (Asendorpf and Baudonniere, 1993), the cognitive capacity to distinguish between emotions, cognitions, and behaviors of one’s self and those of others. This is one mechanism through which employees are made aware of the situation and emotional state of others (Asendorpf and Baudonniere, 1993), and it also enables the recognition of others as salient elements within the environment (Davis and Holtgraves, 1984). Other-awareness involves being conscious of the presence of others as well as their feelings and actions, which is particularly important in enabling the process of noticing the positive emotional experiences of others. Another mechanism that can affect this process is the ability to accurately infer another person’s thoughts or feelings. This capacity to notice changes in emotional expressions has been referred to as empathic accuracy (Ickes, 1993) or emotion recognition ability (Rubin et al., 2005).

Taking notice of the emotional experiences of others can be a simple process or require a degree of effort. When a person explicitly shares their positive experience in a situation of unprompted self-disclosure, the process of noticing is unavoidable and direct. The person can also express clear signs of joy non-verbally, making it easy for the other to recognize their emotional state (Rubin et al., 2005; Côté, 2014). However, in a work context, a colleague’s emotional cues will often be faint and ambiguous and more effort is required from coworkers to consciously observe the positive emotions (Frost, 2003). This effort can be demonstrated by actively attending and listening to coworkers, picking up on behavioral cues, and trusting one’s intuition to understand a colleague’s condition and position (Dutton et al., 2006). Furthermore, the responder can also take an active role in initiating the response, for example, they can inquire about a coworker’s day or their current feelings. The more aware colleagues are of each other’s actions, the more likely they are to actively ask about daily activities and thus facilitate expressions of positive emotions. Initiating a validating response can be particularly powerful when the responder demonstrates an active interest in the coworker’s experiences.

A person’s own mood and conscious prioritizing of positivity may further enable awareness of coworkers’ emotional states. For example, an employee’s state of mind can affect what they notice. A trait study by Devlin et al. (2014) showed that people who were more positive were better at observing positive emotions in others but less skillful at noticing and reacting to negative emotions. Additionally, the experience of being stress-free and unhurried may increase the likelihood of noticing another person’s emotional state. In the famous Good Samaritan experiment (Darley and Batson, 1973), people on their way to give a talk on the parable of the Good Samaritan were less likely to help when they were in a hurry. Moreover, as negative events tend to evoke stronger and more rapid responses than positive or neutral ones Taylor, 1991), people are more prone to noticing negativity and threats than observing safety and positivity (Baumeister et al., 2001). However, people can choose to plan their day to deliberately include situations that lead to an increase in positive emotions (Catalino et al., 2014), and they can also purposefully aim to notice positivity in others. This conscious prioritizing of positivity can act as an antidote and help enable people to notice the cues for positive emotions in their coworkers.

### Making Sense of the Positive Emotions of Others

Following the step of noticing, the responder is then required to make sense of the discloser’s positive emotions. This sensemaking is the interpretive work (Weick, 2012) used by employees to find meaning in ambiguous situations (Weick et al., 2005) so that they can make a decision on the appropriate thoughts and actions. Individuals differ in their ability to understand and analyze emotions (Mayer and Salovey, 1997), but some form of perspective taking is generally required to make sense of the positive emotions of others. Perspective taking refers to a cognitive effort to look at the world from a different point of view or, as defined by Davis (1983: 169), perspective taking is “the spontaneous tendency of the respondent to adopt the psychological perspective of other people – to entertain the point of view of others.” This diverges from social contrasting where the responder focuses more on a comparison between themselves and the other and less on what the other is feeling.

In the effort to make sense of a situation in a way that will lead to perspective taking, it is likely that the responder will aim to understand the positive experience and make predictions about the discloser’s role in the experience, as well as their own role as a responder. According to appraisal theory (Lazarus, 1991), several underlying assumptions can affect the way a responder interprets a situation using their evaluations, assessments, and judgments. In appraisal theory, emotions are viewed as acts of meaning, and the perceiver assigns value or meaning to an object that creates the emotional response (Lazarus, 1991). In social relationships at work, appraisals can affect the ways that social interactions elicit changes in positive emotional states (Kiffin-Petersen et al., 2012). In this paper, the following three appraisals are identified as important for the validating response as they can affect the responder’s engagement in perspective taking: first, the discloser’s deservingness of validation; second, the relevance of the discloser and the discloser’s experience to the responder; and third, the responder’s sense of self-worth.

Appraisals about the discloser’s deservingness relate to “the moral worth of the other” (Clark, 1987: 297). For example, the perceived motive underlying the discloser’s act of expressing their positive emotions may mediate the response of the responder (Duprez et al., 2015; Tamir, 2016). As suggested by Gable et al. (2004: 241), “a positive event may be communicated to share the joy with another, to make a good impression, to establish credibility, to elicit validation, or to increase personal understanding of the situation” – or to incite negative feelings, such as jealousy, if the motives are unkind. Witnessing moral beauty elicits elevation and induces a feeling of warmth toward the person who has elicited the emotion. It can also prompt a willingness to help and a desire to follow the standards of the moral exemplar (Haidt, 2002). Accordingly, the responder is likely to appraise the discloser as more deserving when they perceive them as cooperative, trustworthy, altruistic, or of good character and their motives as positive and sincere. The perceived fairness of the experience in a distributive sense may also affect the responder’s appraisals of the discloser’s deservingness of validation (Ganegoda and Bordia, 2019). The responder’s reaction can be significantly affected if the discloser expresses joy when receiving a reward that the responder feels is undeserved. In contrast, when the reasons for the positive experience conform to implicit norms of allocation, such as equity, equality, and need, then the responder is more likely to interpret the discloser as deserving (Colquitt, 2001) and thus engage in perspective taking.

The responder’s appraisal regarding the relevance of the discloser and their experience relate to how the responder judges the extent to which their own values and goals are congruent or incongruent with the discloser’s positive experience. As suggested by Tesser’s (2000) self-evaluation maintenance model, evaluations will generally lead to differential processes of reflection (“basking in reflected glory”) and comparison (i.e., envy) that further affect the responder’s process of responding. According to this model, changes in self-evaluation are initiated by the outstanding performance of a *close other*. When adapted to the organizational context, *close other* refers to a colleague who is perceived as similar in terms of experience, work attitudes, personality, opinions (Schaubroeck and Lam, 2004), or position held in the organizational structure (Burt, 1987; see also Ganegoda and Bordia, 2019). When a person values or identifies with a performance, they can easily examine the situation from a perspective that uses comparison. If they consider the performance unimportant, the evaluation can be augmented *via* “basking in reflected glory,” that is, feeling that they can also benefit from the good fortune. Thus, people can reduce a threat to self-esteem or augment self-esteem to increase the likelihood of a positive response. This can be achieved by changing the importance of the comparison domain (e.g., identifying less with the performance or making it feel less important), the level of closeness to the comparison person (e.g., perceive the person less similar in terms of experience, work attitudes, personality, opinions, or position), or the performance difference (e.g., performing and the other) (Tesser, 2000). Moreover, another way to trigger perspective taking rather than social comparison is to assume shared group identity with the discloser (e.g., identify a common goal) (Turner, 1975). Studies have shown that shared group identity facilitates perspective taking and increases the likelihood that a person will experience happiness when other people achieve positive outcomes (e.g., Cikara et al., 2014; see also Molenberghs et al., 2014; Ganegoda and Bordia, 2019). Similarly, positive interpersonal relationships and task interdependence help trigger perspective taking rather than social comparison (see Ganegoda and Bordia, 2019). The more someone likes a person, the more motivated they are to take that person’s perspective (McPherson and Janoff-Bulman, 2000), and close interaction among coworkers increases their perspective taking capacity (Parker and Axtell, 2001).

Finally, appraisals regarding self-worth correspond to how secure a person feels when facing the positive experiences of others. Drawing on the research of self-compassion versus self-esteem (Crocker and Park, 2004; Neff and Vonk, 2009), we posit that a more stable sense of self-worth will correlate with a reduction in harmful social comparison and the level of threat perceived from the positive experiences of others. Thus, a person with a strong sense of self-worth should respond to other people’s positive emotions with genuine and respectful validation. Supporting this logic, both self-esteem and self-compassion can equally predict happiness, optimism, and positive affect (Neff and Vonk, 2009). However, when tested in relation to ego-focused reactivity (unhealthy social comparison, such as over-reacting, defensiveness, attacking, or unnecessary fear), self-compassion has been shown to have a stronger negative association with unhealthy social comparison as it is protected by more stable feelings of self-worth. In contrast, self-esteem was positively associated with narcissism. Crocker and Park (2004) explained that while self-esteem does not constitute an unhealthy self-stance, some of the ways that people strive for self-esteem are potentially damaging. In domains, such as work that emphasize self-worth, people aim to validate their self-worth through their abilities or qualities. This can produce unhealthy social comparison or competition and cause a reaction to a threat that undermines connections, such as relatedness, between coworkers. As a result, the situation can challenge a person’s ability to participate in perspective taking and the validation of their coworkers’ positive experiences. For example, when someone shares their positive experience, another person may become preoccupied with the meaning of the experience in relation to their self-worth and this can be detrimental to the process of responding. However, when the responder has a stable sense of self-worth, they are less inclined to use comparisons to validate their own worth and are more inclined to celebrate the success of others.

### Feeling Positive Empathy

A responder will typically feel positive empathy when they use perspective taking to make sense of another person’s positive emotions and thus be able to examine the situation from an alternative point of view (Morelli et al., 2015). Within the workplace, this can be defined as “an employee’s experience of happiness in response to a coworker’s positive experience (state or outcome) and the real or imagined happiness in the coworker” (Ganegoda and Bordia, 2019). Positive empathy is about sharing and enjoying the positive emotions of others (Telle and Pfister, 2016), and this ability can provide the motivational impetus for subsequent validating behavior.

Ganegoda and Bordia (2019) recently examined perspective taking as a sub-process in the context of work, arguing that it provides the typical pathway to feelings of positive empathy. Perspective taking has been shown to successfully decrease prejudice, stereotyping, and social aggression and improve social coordination through increased self-other overlap (Galinsky et al., 2005), thus prompting the responder to share the discloser’s feeling of happiness. Neuropsychological research has also shown that perspective taking triggers positive empathy as they are both activated in the same region of the brain (Morelli et al., 2014).

In addition to perspective taking or cognitive empathy, we recognize affective empathy – direct sharing of the other person’s feelings (Singer and Klimecki, 2014) – as another potential pathway to positive empathy for the responder. This particular pathway can sometimes be influenced by unconscious affective convergence mechanisms, such as primitive emotional contagion and behavioral entrainment (see Hatfield et al., 1994; Collins et al., 2013). Sharing positive emotions has been shown to increase self-other overlap in particular (Waugh and Fredrickson, 2006; Fredrickson, 2013, 2016). It is also strongly correlated with sympathetic caring and largely negatively correlated with meanness (Murphy et al., 2018), resulting in a motivation to invest in the well-being of the other person (Fredrickson, 2013, 2016). Positive empathy is not solely affective or cognitive empathy, but rather representative of an overall empathic concern, described by Batson (1994, p.606) as “other-oriented feelings that are most often congruent with the perceived welfare of the other person.” Positive empathy is initiated and formed through both affective and cognitive understanding of the other person’s positive emotional experience.

Several researchers have argued that positive empathy should motivate people to help others and promote subsequent prosocial behavior (e.g., Morelli et al., 2015; Telle and Pfister, 2016; Ganegoda and Bordia, 2019). Similarly, positive affect has also been associated with increased prosocial motivation and behavior (McCullough et al., 2002; Lyubomirsky et al., 2005) as well as empathy for others (e.g., McCullough et al., 2002), and these have been further linked to several important organizational outcomes (Clark et al., 2018). Thus, a person experiencing positive empathy is more likely to behave in helpful and validating ways toward the other.

### Acting to Validate the Positive Emotions of Others

Acting is the sub-process that captures all the behaviors of the responder that can validate the positive emotional experiences of the discloser in order to effect symmetrical emotion regulation outcomes. In emotionally loaded situations, people may choose to employ different emotion regulation strategies such as reappraisal or distraction (Sheppes et al., 2011), which help them to achieve their desired outcomes (Côté et al., 2011). In here, we will focus on two behavioral mechanisms that are likely to promote a validating response. In relationship studies, responsiveness is viewed as a key component of a dyadic interaction in which the responder communicates understanding, validation, and caring in response to another person’s self-disclosure (Reis and Shaver, 1988). Responsiveness refers to the means and extent to which the responder conveys validation by addressing the discloser’s actions, communications, needs, and wants from the previous interaction (Davis, 1982; Miller and Berg, 1984; Berg, 1987). To contribute positively to the development of the relationship, responsive behaviors need to be sincere and demonstrate concern and caring for the discloser, as well as capture the content of the discloser’s original communication (Berg, 1987).

Research on capitalization by Gable et al. (2004) studied the responsive behavior from the perspective of the discloser and identified four ways that a responder can respond to a discloser’s positive news: active-constructive (enthusiastic encouragement, e.g., “wonderful, let us celebrate”) or active-unconstructive (quashing the news, e.g., “that’s not enough”) and passive-constructive (understated support, e.g., one is present, but does not say anything positive) or passive-unconstructive (ignoring the news, e.g., one looks at their phone instead of the person telling the news). In terms of positive outcomes, only the perceived active-constructive response is beneficial as it refers to acts that promote feelings of understanding, validation, and caring. With an active-constructive response, the responder can indicate that they understand something that is central to the discloser, such as a goal attainment, a positive affect, or a meaningful activity that is personally relevant, whereas with the other three responses the responder fails to indicate such understanding (Gable et al., 2004; Gable and Reis, 2010). The three other types of responses were negatively associated with the amount of positive emotions experienced and the perceived quality of the relationship (Gable et al., 2004).

In organizational studies, respectful engagement has been recognized as one of the key behavioral mechanisms involved in building high-quality social connections between employees in a workplace (Stephens et al., 2011). Defined as behaviors that show esteem, dignity, and care for another person (e.g., Ramarajan et al., 2008), respectful engagement should be viewed as a collection of behaviors that enable a validating response from the responder. These behaviors cover a broad range and include common, frequent, often small, and sometimes subconscious gestures, words, tones, and body postures that communicate the level of respect for the other person and their perceived value (Stephens et al., 2011). The behaviors also extend to more conscious actions that communicate affirmation of the discloser’s worth and value, such as a caring presence (Kahn, 1992) and expressions of genuine gratitude (Grant and Gino, 2010). By demonstrating the human need for respect and dignity (Rawls, 1971), these behaviors should assist with the formation of a response that addresses the discloser and their emotional experience in a validating way.

While research on capitalization suggests that enthusiastic responses are the key to positive outcomes (e.g., Reis et al., 2010), this article emphasizes the importance of a behavioral response that is genuine and signals validation of the discloser and their current state of happiness. Overall, positive emotions can be strengthened and their related resources increased through acts of savoring and exploring, such as elaboration, repetition, retelling, and rehearsing (Fredrickson, 1998, 2001, 2004; Fredrickson and Joiner, 2002). Accordingly, actions that help the discloser savor their positive emotions are beneficial as they can strengthen the positive memory of the experience. For example, a responder can ask a disclosure to elaborate on their thoughts and feelings regarding a positive emotional experience. This action, in turn, can lead to increased well-being and better relationship quality (Gable et al., 2004). Specifically, responsive and respectful validation of the discloser’s experience may help amplify positive emotions.

### Potential Outcomes

As an affective phenomenon marked by positive emotions, the process of responding to validate coworkers’ positive emotions has important implications in the workplace. At intrapersonal, interpersonal, and team levels, we identify four significant sets of outcomes: well-being and creativity, high-quality connections and collaboration, interpersonal capitalization and prosocial behavior, and psychological safety, and team learning.

At the intrapersonal level, a successful validating response to another person’s positive event has been shown to increase the amount of positive affect for both the responder (Monfort et al., 2014; Conoley et al., 2015) and the discloser (Gable et al., 2004; Bryant et al., 2005; Maisel and Gable, 2009). Although positive events and positive emotions are different, they can share considerable overlap in terms of the potential impacts on consequential positive affect; both processes require a validating response to the positive emotional experience of the discloser that is connected to the shared positive event or the expressed positive emotion. Additionally, we posit that through emotional contagion (Hatfield et al., 1994), the positive affect that is generated can potentially extend to third parties witnessing the response. Research has provided substantial evidence that positive emotions have positive impacts on an individual’s well-being and creativity (e.g., Amabile et al., 2005; Lyubomirsky et al., 2005), and examples have been provided by the broaden and build theory of positive emotions (Fredrickson, 1998, 2001, 2004; Fredrickson et al., 2008) and the recent empirical studies testing this theory (Major et al., 2018). Therefore, we expect that the validating response to a coworker’s positive emotions positively impacts on the well-being and creativity of the witnesses and the individuals taking part in the unfolding of the response. Moreover, as well as feeling the positive emotions, expressing them may also be important in terms of positive health and well-being outcomes. For example, when participants in a study watched a happy video, their immune system showed increased activity; however, this effect was only demonstrated when they were instructed to express their emotions (Labott et al., 1990). Thus, when the expression of positive emotions is encouraged, the validating response should contribute positively to well-being.

At the interpersonal level, a validating response to the positive emotions of others is likely to have a positive impact on the quality of interpersonal connections, relationships, collaboration, and trust. Research suggests that when an employee responds to a coworker’s positive experience with positive empathy, the interpersonal relationship between them is strengthened through feelings of affection and warmth (Hareli and Rafaeli, 2008; Morelli et al., 2015). Similarly, a successful validation of a discloser’s positive event benefits the perceived quality of the relationship (e.g., Peters et al., 2018). Moreover, an employee can experience feelings of gratitude when they perceive a coworker intentionally contributing something of value, such as providing a validating response to another person’s emotions (e.g., Emmons and Shelton, 2001; Fredrickson, 2004). When one person feels gratitude, both members of an interaction can experience a greater connection, and this can also develop further over time (Algoe et al., 2008). In turn, higher quality connections between employees enhance collaboration and trust among coworkers (e.g., Dutton and Heaphy, 2003; Stephens et al., 2011). Additionally, positive affect directly produces desirable effects for collaborative work behaviors by decreasing conflicts and enhancing willingness to collaborate (Barsade, 2002). More generally, the broadening effect of positive emotions helps to build durable social resources (Fredrickson, 1998, 2001). This includes the development of increased closeness in relationships in terms of self-other overlap, which can also boost the feeling of connection quality even in new relationships (Waugh and Fredrickson, 2006). People who generally report greater positive affect demonstrate better social skills and have interactions of higher quality (Berry and Hansen, 1996).

The validating response is also likely to foster interpersonal capitalization and prosocial behavior that create a beneficial cycle of positive affect and prosocial relationships. As suggested by Peters et al. (2018), successful capitalization efforts should promote further attempts. In particular, future interpersonal capitalization is encouraged when the positive emotion of gratitude creates a mutually rewarding cycle of positive affect through increased prosocial activity and empathy for others (e.g., McCullough et al., 2002). Witnessing or experiencing the responder’s positive empathy and related prosocial motivation will generally reduce a person’s fear of receiving a dampening response to their positive emotions, thus enabling further interpersonal capitalization attempts (Hadley, 2014). Positive affect is the integral mechanism and outcome of interpersonal capitalization, and it has been consistently linked to prosocial behavior (e.g., Isen et al., 1976; Carlson et al., 1988; Baron, 1997; Aknin et al., 2012). In particular, those experiencing positive empathy are more likely to engage in prosocial behavior (Andreychik and Migliaccio, 2015; Morelli et al., 2015) and Telle and Pfister (2016) suggested that people do so in order to maintain their positive affect. In addition to high-quality connections and collaboration at the interpersonal level, the validating response should thus generate prosocial behavior among colleagues through positive cycles of increased positive affect and interpersonal capitalization.

At the team level, this article proposes that the validating response contributes positively to employees’ feelings of psychological safety and thus enhances, for instance, team learning. Increased positive affect promotes the disclosure of information to others (Fredrickson, 1998, 2001), and it encourages interaction rather than characteristics of poor psychological safety (Kahn, 1990), such as withdrawn or defensive behavior (Waugh and Fredrickson, 2006). Furthermore, witnessing a colleague responding positively to a coworker’s needs and emotions through a validating response is likely to decrease an employee’s negative perceptions of the consequences of taking interpersonal risks (Edmondson, 1999) and thus in turn encourage them to express themselves willingly (Berg, 1987). Such experiences involve the key elements of psychological safety and team learning (Kahn, 1990; Edmondson, 1999; Edmondson and Lei, 2014). For example, engaging respectfully with a coworker fosters acceptance and openness and motivates interaction and sharing (Carmeli et al., 2015). When validation occurs, it produces feelings of being understood and cared for (Davis, 1982; Berg, 1987) which in turn contributes to building a sense of safety (Reis and Shaver, 1988; Edmondson, 1999). This contrasts with the anticipation of rejection or judgment that stops people from expressing and sharing their emotional experiences (Gable et al., 2004; Maisel and Gable, 2009). This article asserts that validating responses build psychological safety and thus enhance also team learning by decreasing the focus on the negative consequences of taking interpersonal risks and increasing employees’ willingness to express themselves.

### Boundary Conditions and Contextual Factors

While we have restricted our focus to the four steps of the responder’s validating response and their related mechanisms, the responding process is inevitably affected by numerous contextual attributes and conditions at the personal and organizational levels that also influence the resulting outcomes. The first key role that should be examined is the position of the discloser in the responding process. A triggered positive emotional experience often conveys information about the associated emotions, values, or needs of the discloser (Gable and Reis, 2010); therefore, expressing positivity can make a person feel vulnerable and reluctant to share their thoughts and feelings. This can be particularly evident when the discloser resists the expression of positive emotions due to the anticipation of rejection, judgment, or defensiveness (Gable et al., 2004; Maisel and Gable, 2009). However, the process of responding will not unfold if the discloser fails to express their positive emotions in some way. People can also quickly read gestures and facial expressions to make rapid judgments on whether others are accepting and warm (Ambady et al., 2000), and this is likely to shape whether or not a person chooses to express their emotions. Thus, the discloser’s willingness to share positive emotions can significantly influence the initiation of the validating process.

Furthermore, the type of positive emotions the discloser shares can shape the response. Different kinds of positive emotions can cause a variety of reactions that shape the responder’s sensemaking and response. For example, a person might be comfortable with achievement-based positive affect but not affiliative or vice versa (Gilbert et al., 2014). Consequently, for a responder who is uncomfortable with affiliative affect, its expressions would cause distress and hinder or block positive empathy and the validating response (Gilbert et al., 2014).

The personal qualities of the responder also influence the process. For example, the trait of positive empathy at the personal level (Morelli et al., 2015) is affected by an individual’s dispositional capacity for cognitive perspective taking (Andreychik and Migliaccio, 2015) as well as their tendency to view themselves in terms of their relationships with others, that is, interdependent self-construal (Galinsky et al., 2005; Varnum et al., 2014). Equally, positive empathy is influenced by an individual’s propensity to experience positive emotions, that is, positive affectivity (Sallquist et al., 2009; Morelli et al., 2015). Higher levels of positive affectivity can trigger perspective taking and thus affect a person’s response to their coworkers’ positive emotions (see Ganegoda and Bordia, 2019).

At the organizational level, shared values, norms, and practices signal what is appropriate, valued, or encouraged, as well as the type of emotional expressions and responses that are expected (see Dutton et al., 2014). For example, shared values sensitize an employee’s tendency to notice certain situations or actions (Dutton et al., 2006), and organizational norms regarding feelings influence how and when certain emotions are expressed at work (Hochschild, 1979). When it comes to directing attentional energy in organizations, people in positions of authority are particularly influential as employees pay more attention to their leaders’ behavior than vice versa (e.g., Worline and Dutton, 2017). A leader’s own example and encouragement to react in a positive way can significantly influence how easy or difficult it is to express positive emotions in the workplace. Through their own way of reacting to others’ positive emotions, leaders provide a model for their staff on how such situations should be approached in the organization.

## Discussion

This article has provided a theoretical and conceptual exploration of employees’ positive responses to coworkers’ positive emotional experiences, introduced here as a validating response. We have underscored the importance of the validating response in the realm of interpersonal dynamics in the workplace and suggested a four-step model for the responder that includes noticing, sensemaking, feeling positive empathy, and engaging in validating behavior. For each of the four steps, we have identified key mechanisms that facilitate the validating response. We have further connected employees’ validating responses to particularly important potential individual and organizational outcomes and recognized the contextual factors that enhance or impede the validating response.

By studying the validating response, this article makes a contribution to three important research streams on positive interpersonal responding, namely, interpersonal capitalization, psychological safety, and high-quality connections. First, concerning interpersonal capitalization and emotion regulation outcomes, we have identified key steps and mechanisms that move the perspective of the responder to the forefront of the research. By studying this previously overlooked perspective, we can now better address how a validating response to the positive emotions of others can bring about desired symmetrical emotion regulation outcomes. This new framework calls attention to the perspective of the responder and expands the focus from responding to positive events to also responding to positive emotions. By proposing that positive empathy is analogous to the role of empathic concern in the process of compassion (Dutton et al., 2014), the current research expands the theory and research on positive empathy, suggesting that it presents one key part of the unfolding of the process of a validating response, but not the entire response, as suggested by previous research (Ganegoda and Bordia, 2019).

Second, our work addresses the understudied question of how psychological safety is built and protected in organizations (Edmondson and Lei, 2014). Anticipating a response that lacks validation is an interpersonal risk that can undermine psychological safety within teams and lead to less sharing of ideas, questions, and feedback (Edmondson, 1999). Receiving a validating response protects and builds psychological safety by decreasing interpersonal risk and increasing employees’ willingness to express themselves. William Kahn (1990) observed that when interactions demonstrate respect and trust – both key characteristics of validating behavior – people are more likely to anticipate a genuine response and assume the situation involves less interpersonal risk – a defining characteristic of psychological safety. The expression of a person’s positive emotions is an example of an action that carries this interpersonal risk (Hadley, 2014). Psychological safety is generally built over time through positive responses to such displays of interpersonal risk. The validating response can enhance psychological safety and team learning by prompting uninhibited communication, decreasing negative perceptions of the consequences of interpersonal risks, fostering acceptance and openness, and motivating interaction and sharing among colleagues.

Third, HQCs have been associated with improved collaboration, relationship quality, and trust among colleagues (e.g., Dutton and Heaphy, 2003; Stephens et al., 2011). Our paper has addressed a gap in this research by framing the validating response in a model that illustrates a specific approach to creating HQCs. We have also extended the understanding of what enables HQCs by identifying the different cognitive, emotional, and behavioral mechanisms and their interdependence. Particularly, our analysis has identified that in terms of constructing HQCs through the validating response, other-awareness precedes perspective taking. Perspective taking, empathy, and emotional contagion then precede respectful engagement. Our model has also highlighted positive emotions as the underlying mechanism that facilitates a person’s acceptance of interactions and generates the positive outcomes of the validating response. However, positive emotions do not necessarily lead directly to HQCs as they are dependent on social interactions and the ways in which they are received and validated by the responder. This paper has also introduced positive empathy as the critical mechanism that enables the validating response that, in turn, facilitates the development of HQCs.

### Practical Implications and Leverage Points for Organizations

The process approach to a validating response uncovers multiple ways for employees to exercise agency to foster positive responses to coworkers’ positive experiences. Developing these responses can promote well-being, quality connections, collaboration, and team learning. Each step of the model has mechanisms that employees can practice to shape their behavior and their work environments. First, for example, mindfulness practice may help employees improve their ability to notice coworkers’ positive emotions through better other-awareness and empathy (Krasner et al., 2009; Atkins, 2013). Second, employees can actively practice perspective taking to foster sensemaking of other people and their experiences so they can be viewed as fair and deserving of validation; practicing self-compassion can also address harmful social comparisons (Neff and Vonk, 2009). Third, employees may intentionally cultivate a capacity to celebrate and enjoy their coworkers’ positive emotions through practices such as loving-kindness meditation (Fredrickson et al., 2008).

Fourth, employees can experiment with different ways of showing respect and demonstrating responsiveness, for example, through active listening, follow-up questions, and genuine interest in the activities of others. There are also many contextual factors that have an impact on the validation process that leaders can use to shape the working conditions in a way that supports the expression of positive emotional experiences and the forms of their validation. For example, leaders can use organizational incentives and practices as well as constructive behaviors to encourage and demonstrate values such as cooperation, prosocial behavior, admission of mistakes, and requests for help. Using these methods, leaders can help direct employees to anticipate less interpersonal risk when sharing and responding to each other’s positive emotions.

### Directions for Future Research

The theoretical analysis of the current paper offers a number of avenues for future research. A central limitation of this study is the lack of empirical testing of the model created in this paper. Qualitative diary and interview methods could be used to increase the understanding of the perceived experiences of the responder. Also, data that are not reliant on self-report could be generated both through ethnography at workplaces as well as through objective measures of observation or laboratory studies. In particular, questionnaire measures for each step of the responding process should be developed to further identify the roles of each step and their mechanisms in the validating response process. Experimental studies, such as intervention studies, should also be completed in organizations, to reveal how to increase the responsiveness of responders and the potential impacts if the increase is possible. Dyads of discloser and responder could simultaneously be observed in laboratory setting to understand dynamics such as body language and unconscious messaging that affect the validation process in real time.

One particular angle of a lacunae in research concerns the responder viewpoint. We have introduced the responder as the potential initiator of the responding process and addressed the need to examine interpersonal capitalization in the organizational context. To date, organizational research has not considered the organizational outcomes of responding to coworkers’ positive emotions in a validating way. Therefore, we suggest that future research should empirically examine the potentially significant impacts of these responses at the intrapersonal, interpersonal, and team levels.

Moreover, our research has identified the similarities and differences between the two interpersonal processes of responding to the emotional experiences of others, namely, compassion as a response to the negative emotions of the other and the validating response as a response to the positive emotions of the other. However, further research is required to better understand how they are interrelated and how they might affect each other.

### Conclusion

In today’s contradictory work life, employees require ever more cognitive endurance and interpersonal skills. These skills are inevitably affected by emotions as well as how the emotions are expressed and received in the workplace. Driven by performance and efficiency, organizations continue to maintain achievement-oriented cultures that depreciate emotions and produce fatigue and disengagement among employees. A validating response to a coworker’s positive emotions may act as an antidote to a negative environment, and the positive implications are likely to also influence lives outside the workplace. This investigation presents a new and significant perspective to promoting high-quality interpersonal relating and desired emotion regulation outcomes in organizations through the validating response. The theoretical model developed in this article provides a starting point for further work on positive interrelating that acknowledges the synergy between compassion and the validating response.

## Data Availability Statement

The original contributions presented in the study are included in the article/supplementary material, further inquiries can be directed to the corresponding author.

## Author Contributions

MP, FM, and AP contributed to the conception of the study. MP wrote the first draft of the article. FM and AP commented and helped to revise the first drafts. MP finalized the paper. All authors read and approved the submitted version.

## Funding

The Finnish Work Environment Fund (Työsuojelurahasto), 180164, funding for dissertation research Helsinki University Library (Helsingin yliopiston kirjasto), funding for open access publication fees.

## Conflict of Interest

The authors declare that the research was conducted in the absence of any commercial or financial relationships that could be construed as a potential conflict of interest.

## Publisher’s Note

All claims expressed in this article are solely those of the authors and do not necessarily represent those of their affiliated organizations, or those of the publisher, the editors and the reviewers. Any product that may be evaluated in this article, or claim that may be made by its manufacturer, is not guaranteed or endorsed by the publisher.

## References

[ref2] Aknin L. Dunn E. Norton M. (2012). Happiness runs in a circular motion: evidence for a positive feedback loop between prosocial spending and happiness. J. Happiness Stud. 13, 347–355. doi: 10.1007/s10902-011-9267-5

[ref3] Algoe S. B. Haidt J. Gable S. L. (2008). Beyond reciprocity: gratitude and relationships in everyday life. Emotion 8, 425–429. doi: 10.1037/1528-3542.8.3.425 18540759 PMC2692821

[ref4] Amabile T. M. Barsade S. G. Mueller J. S. Staw B. M. (2005). Affect and creativity at work. Adm. Sci. Q. 50, 367–403. doi: 10.2189/asqu.2005.50.3.367

[ref5] Amabile T. M. Kramer S. J. (2007). Inner work life: understanding the subtext of business performance. Harv. Bus. Rev. 85, 72–86. doi: 10.1109/EMR.2015.7059374, PMID: 17494252

[ref6] Ambady N. Bernieri F. J. Richeson J. A. (2000). “Toward a histology of social behavior: judgmental accuracy from thin slices of the behavioral stream,” in Advances in Experimental Social Psychology, 32. ed. Zanna M. P. (San Diego: Academic Press), 201–271.

[ref7] Andreychik M. R. Migliaccio N. (2015). Empathizing with others’ pain versus empathizing with others’ joy: examining the separability of positive and negative empathy and their relation to different types of social behaviors and social emotions. Basic Appl. Soc. Psychol. 37, 274–291. doi: 10.1080/01973533.2015.1071256

[ref8] Asendorpf J. B. Baudonniere P. M. (1993). Self-awareness and other-awareness: Mirror self-recognition and synchronic imitation among unfamiliar peers. Dev. Psychol. 29, 88–95. doi: 10.1037/0012-1649.29.1.88

[ref9] Ashforth B. E. Humphrey R. H. (1995). Emotion in the workplace: A reappraisal. Hum. Relat. 48, 97–125. doi: 10.1177/001872679504800201

[ref10] Atkins P. W. B. (2013). “Empathy, self-other differentiation and mindfulness,” in Organizing Through Empathy. eds. Pavlovich K. Krahnke K. (New York: Routledge), 49–70.

[ref11] Baron R. A. (1997). The sweet smell of… helping: effects of pleasant ambient fragrance on prosocial behavior in shopping malls. Personal. Soc. Psychol. Bull. 23, 498–503. doi: 10.1177/0146167297235005

[ref12] Barsade S. G. (2002). The ripple effect: emotional contagion and its influence on group behavior. Adm. Sci. Q. 47, 644–675. doi: 10.2307/3094912

[ref13] Batson C. D. (1994). Why act for the public good? Four answers. Personal. Soc. Psychol. Bull. 20, 603–610. doi: 10.1177/0146167294205016

[ref14] Baumeister R. F. Bratslavsky E. Finkenauer C. Vohs K. D. (2001). Bad is stronger than good. Rev. Gen. Psychol. 5, 323. doi: 10.1037/1089-2680.5.4.323

[ref15] Baumeister R. F. Leary M. R. (1995). The need to belong: desire for interpersonal attachments as a fundamental human motivation. Psychol. Bull. 117, 497–529. doi: 10.1037/0033-2909.117.3.497, PMID: 7777651

[ref16] Berg J. H. (1987). “Responsiveness and self-disclosure,” in Self-Disclosure: Theory, Research, and Therapy. eds. Derlega V. J. Berg J. H. (New York: Plenum), 101–130.

[ref17] Berry D. Hansen J. (1996). Positive affect, negative affect, and social interaction. J. Pers. Soc. Psychol. 71, 796–809. doi: 10.1037/0022-3514.71.4.796

[ref18] Brief A. P. Weiss H. M. (2002). Organizational behavior: affect in the workplace. Annu. Rev. Psychol. 53, 279–308. doi: 10.1146/annurev.psych.53.100901.135156, PMID: 11752487

[ref19] Bryant F. B. Smart C. M. King S. P. (2005). Using the past to enhance the present: boosting happiness through positive reminiscence. J. Happiness Stud. 6, 227–260. doi: 10.1007/s10902-005-3889-4

[ref20] Burt R. (1987). Social contagion and innovation: cohesion versus structural equivalence. Am. J. Sociol. 92, 1287–1335. doi: 10.1086/228667

[ref21] Cacioppo J. T. Gardner W. L. Berntson G. G. (1997). Beyond bipolar conceptualizations and measures: The case of attitudes and evaluative space. Personal. Soc. Psychol. Rev. 1, 3–25. doi: 10.1207/s15327957pspr0101_2, PMID: 15647126

[ref22] Carlson M. Charlin V. Miller N. (1988). Positive mood and helping behavior: A test of six hypotheses. J. Pers. Soc. Psychol. 55, 211–229. doi: 10.1037/0022-3514.55.2.211, PMID: 3050025

[ref23] Carmeli A. Dutton J. Hardin A. (2015). Respect as an engine for new ideas: linking respectful engagement, relational information processing and creativity among employees and teams. Hum. Relat. 68, 1021–1047. doi: 10.1177/0018726714550256

[ref24] Catalino L. Algoe S. Fredrickson B. (2014). Prioritizing positivity: An affective approach to pursuing happiness? Emotion 14, 1155–1161. doi: 10.1037/a0038029, PMID: 25401290 PMC5533095

[ref25] Cikara M. Bruneau E. Van Bavel J. Saxe R. (2014). Their pain gives us pleasure: how intergroup dynamics shape empathic failures and counter-empathic responses. J. Exp. Soc. Psychol. 55, 110–125. doi: 10.1016/j.jesp.2014.06.007, PMID: 25082998 PMC4112600

[ref26] Clark C. (1987). Sympathy biography and sympathy margin. Am. J. Sociol. 93, 290–321. doi: 10.1086/228746

[ref27] Clark M. Robertson M. Young S. (2018). “I feel your pain:” A critical review of organizational research on empathy. J. Organ. Behav. 40, 166–192. doi: 10.1002/job.2348

[ref01] Collins A. L. Lawrence S. A. Troth A. C. Jordan P. J. (2013). Group affective tone: A review and future research directions. J. Organ. Behav. 34, S43–S62. doi: 10.1002/job.1887

[ref28] Colquitt J. (2001). On the dimensionality of organizational justice: A construct validation of a measure. J. Appl. Psychol. 86, 386–400. doi: 10.1037/0021-9010.86.3.386 11419799

[ref29] Conoley C. Vasquez E. Del Carmen B. Oromendia M. Jeske D. (2015). Celebrating the accomplishments of others: mutual benefits of capitalization. Couns. Psychol. 43, 734–751. doi: 10.1177/0011000015584066

[ref30] Côté S. (2014). Emotional intelligence in organizations. Annu. Rev. Organ. Psychol. Organ. Behav. 1, 459–488. doi: 10.1146/annurev-orgpsych-031413-091233

[ref31] Côté S. DeCelles K. A. McCarthy J. M. Van Kleef G. A. Hideg I. (2011). The Jekyll and Hyde of emotional intelligence: emotion-regulation knowledge facilitates both prosocial and interpersonally deviant behavior. Psychol. Sci. 22, 1073–1080. doi: 10.1177/0956797611416251, PMID: 21775654

[ref32] Crocker J. Park L. (2004). The costly pursuit of self-esteem. Psychol. Bull. 130, 392–414. doi: 10.1037/0033-2909.130.3.392, PMID: 15122925

[ref33] Darley J. Batson D. (1973). From Jerusalem to Jericho: A study of situational and dispositional variables in helping behavior. J. Pers. Soc. Psychol. 27, 100–108. doi: 10.1037/h0034449

[ref34] Davis D. (1982). “Determinants of responsiveness in dyadic interaction,” in Personality, Roles, and Social Behaviors. eds. Ickes W. Knowles E. (New York: Springer-Verlag), 85–139.

[ref35] Davis M. (1983). The effects of dispositional empathy on emotional reactions and helping: A multidimensional approach. J. Pers. 51, 167–184. doi: 10.1111/j.1467-6494.1983.tb00860.x

[ref36] Davis D. Holtgraves T. (1984). Perceptions of unresponsive others: attributions, attraction, understandability and memory of their utterances. J. Exp. Soc. Psychol. 20, 383–408. doi: 10.1016/0022-1031(84)90034-9

[ref37] Deci E. Ryan R. (2000). The “what” and “why” of goal pursuits: human needs and the self-determination of behavior. Psychol. Inq. 11, 227–268. doi: 10.1207/S15327965PLI1104_01

[ref38] Devlin H. Zaki J. Ong D. Gruber J. (2014). Not as good as you think? Trait positive emotions is associated with increased self-reported empathy but decreased empathic performance. PLoS One 9, 1–8. doi: 10.1371/journal.pone.0110470 PMC4212943 25353635

[ref39] Duffy M. Scott K. Shaw J. Tepper B. Aquino K. (2012). A social context model of envy and social undermining. Acad. Manag. J. 55, 643–666. doi: 10.5465/amj.2009.0804

[ref40] Duprez C. Christophe V. Rimé B. Congard A. Antoine P. (2015). Motives for the social sharing of an emotional experience. J. Soc. Pers. Relat. 32, 757–787. doi: 10.1177/0265407514548393

[ref41] Dutton J. (2003). Breathing life into organizational studies. J. Manag. Inq. 12, 5–19. doi: 10.1177/1056492602250515

[ref42] Dutton J. Heaphy E. (2003). “The power of high-quality connections,” in Positive Organizational Scholarship: Foundations of a New Discipline. eds. Cameron K. Dutton J. Quinn R. (San Francisco: Berrett-Koehler), 263–278.

[ref43] Dutton J. Workman K. Hardin A. (2014). Compassion at work. Annu. Rev. Organ. Psych. Organ. Behav. 1, 277–304. doi: 10.1146/annurev-orgpsych-031413-091221

[ref44] Dutton J. Worline M. Frost P. Lilius J. (2006). Explaining compassion organizing. Adm. Sci. Q. 51, 59–96. doi: 10.2189/asqu.51.1.59

[ref45] Edmondson A. (1999). Psychological safety and learning behavior in work teams. Adm. Sci. Q. 44, 350–383. doi: 10.2307/2666999

[ref46] Edmondson A. (2003). Speaking up in the operating room: how team leaders promote learning in interdisciplinary action teams. J. Manag. Stud. 40, 1419–1452. doi: 10.1111/1467-6486.00386

[ref47] Edmondson A. Lei Z. (2014). Psychological safety: The history, renaissance, and future of an interpersonal construct. Annu. Rev. Organ. Psychol. Organ. Behav. 1, 23–43. doi: 10.1146/annurev-orgpsych-031413-091305

[ref48] Eisenberger N. Lieberman M. Williams K. (2003). Does rejection hurt? An fMRI study of social exclusion. Science 302, 290–292. doi: 10.1126/science.1089134, PMID: 14551436

[ref49] Emmons R. Shelton C. (2001). “Gratitude and the science of positive psychology,” in Oxford Handbook of Positive Psychology. eds. Snyder C. Lopez S. (New York: Oxford University Press), 459–471.

[ref50] Frazier M. Fainshmidt S. Klinger R. Pezeshkan A. Vracheva V. (2017). Psychological safety: A meta-analytic review and extension. Pers. Psychol. 70, 113–165. doi: 10.1111/peps.12183

[ref51] Fredrickson B. (1998). What good are positive emotions? Rev. Gen. Psychol. 2, 300–319. doi: 10.1037/1089-2680.2.3.300, PMID: 21850154 PMC3156001

[ref52] Fredrickson B. (2001). The role of positive emotions in positive psychology: The broaden-and-build theory of positive emotions. Am. Psychol. 56, 218–226. doi: 10.1037/0003-066X.56.3.218 11315248 PMC3122271

[ref53] Fredrickson B. (2004). The broaden-and-build theory of positive emotions. Philos. Trans. R. Soc. 359, 1367–1378. doi: 10.1098/rstb.2004.1512, PMID: 15347528 PMC1693418

[ref54] Fredrickson B. (2013). Love2.0.: How our Supreme Emotion Affects Everything we Feel, Think, Do, and Become. New York: Penguin Group.

[ref55] Fredrickson B. (2016). “Love: positivity resonance as a fresh, evidence-based perspective on an age-old topic,” in Handbook of Emotions. eds. Barrett L. Haviland J. 4th *Edn*. (New York, NY: Guilford Press), 847–858.

[ref56] Fredrickson B. Cohn M. Coffey K. Pek J. Finkel S. (2008). Open hearts build lives: positive emotions, induced through loving-kindness meditation, build consequential personal resources. J. Pers. Soc. Psychol. 95, 1045–1062. doi: 10.1037/a0013262, PMID: 18954193 PMC3156028

[ref57] Fredrickson B. Joiner T. (2002). Positive emotions trigger upward spirals toward emotional well-being. Psychol. Sci. 13, 172–175. doi: 10.1111/1467-9280.00431, PMID: 11934003

[ref58] Frost P. (2003). Toxic Emotions at Work: How Compassionate Managers Handle Pain and Conflict. Boston: Harvard Business School Press.

[ref59] Gable S. Reis H. (2010). Good news! Capitalization on positive events in an interpersonal context. Adv. Exp. Soc. Psychol. 42, 195–257. doi: 10.1016/S0065-2601(10)42004-3

[ref60] Gable S. Reis H. Impett E. Asher E. (2004). What do you do when things go right? The intrapersonal and interpersonal benefits of sharing positive events. J. Pers. Soc. Psychol. 87, 228–245. doi: 10.1037/0022-3514.87.2.228, PMID: 15301629

[ref61] Galinsky A. Ku G. Wang C. (2005). Perspective-taking and self-other overlap: fostering social bonds and facilitating social coordination. Group Process. Intergroup Relat. 8, 109–124. doi: 10.1177/1368430205051060

[ref62] Ganegoda D. Bordia P. (2019). I can be happy for you, but not all the time: A contingency model of envy and positive empathy in the workplace. J. Appl. Psychol. 104, 776–795. doi: 10.1037/apl0000377 30556706

[ref63] Gilbert P. McEwan K. Catarino F. Baiao R. Palmeira L. (2014). Fears of happiness and compassion in relationships with depression, alexithymia, and attachment security in a depressed sample. Br. J. Clin. Psychol. 53, 228–244. doi: 10.1111/bjc.12037 24283291

[ref64] Hadley C. (2014). Emotional roulette? Symmetrical and asymmetrical emotion regulation outcomes from coworker interactions about positive and negative work events. Hum. Relat. 67, 1073–1094. doi: 10.1177/0018726714529316

[ref65] Haidt J. (2002). “The moral emotions,” in Handbook of Affective Sciences. eds. Davidson R. J. Scherer K. R. Goldsmith H. H. (New York: Oxford University Press), 852–870.

[ref66] Hareli S. Rafaeli A. (2008). Emotions cycles: On the social influence of emotion in organizations. Res. Organ. Behav. 28, 35–59. doi: 10.1016/j.riob.2008.04.007

[ref67] Hatfield E. Cacioppo J. Rapson R. (1994). Emotional contagion. New York, NY: Cambridge University Press.

[ref68] Hochschild A. (1979). Emotion work, feeling rules, and social structure. Am. J. Sociol. 85, 551–575. doi: 10.1086/227049

[ref69] Ickes W. (1993). Empathic accuracy. J. Pers. 61, 587–610. doi: 10.1111/j.1467-6494.1993.tb00783.x

[ref70] Isen A. Clark M. Schwartz M. (1976). Duration of the effect of good mood on helping: “Fooprints on the sands of time.” J. Pers. Soc. Psychol. 34, 385–393. doi: 10.1037/0022-3514.34.3.385

[ref71] Kahn W. (1990). Psychological conditions of personal engagement and disengagement at work. Acad. Manag. J. 33, 692–724. doi: 10.2307/256287

[ref72] Kahn W. (1992). To be fully there: psychological presence at work. Hum. Relat. 45, 321–349. doi: 10.1177/001872679204500402

[ref73] Kanov J. Maitlis S. Worline M. Dutton J. Frost P. Lilius J. (2004). Compassion in organizational life. American Behavioral. Science 47, 808–827. doi: 10.1177/0002764203260211

[ref74] Kashdan T. Ferssizidis P. Farmer A. Adams L. McKnight P. (2013). Failure to capitalize on sharing good news with romantic partners: exploring positivity deficits of socially anxious people with self-reports, partner-reports, and behavioral observations. Behav. Res. Ther. 51, 656–668. doi: 10.1016/j.brat.2013.04.006, PMID: 23916635 PMC3776926

[ref75] Kiffin-Petersen S. Murphy S. Soutar G. (2012). The problem-solving service worker: appraisal mechanisms and positive affective experiences during customer interactions. Hum. Relat. 65, 1179–1206. doi: 10.1177/0018726712451762

[ref76] Krasner M. S. Epstein R. M. Beckman H. Suchman A. L. Chapman B. Mooney C. J.. (2009). Association of an educational program in mindful communication with burnout, empathy, and attitudes among primary care physicians. JAMA 302, 1284–1293. doi: 10.1001/jama.2009.1384, PMID: 19773563

[ref77] Labott S. Ahleman S. Wolever M. Martin R. (1990). The physiological and psychological effects of the expression and inhibition of emotion. Behav. Med. 16, 182–189. doi: 10.1080/08964289.1990.9934608, PMID: 2271804

[ref78] Langston C. (1994). Capitalizing on and coping with daily life events: expressive responses to positive events. J. Pers. Soc. Psychol. 67, 1112–1125. doi: 10.1037/0022-3514.67.6.1112

[ref79] Lazarus R. (1991). Emotion and Adaptation. New York: Oxford University Press.

[ref80] Lilius J. Worline M. Maitlis S. Kanov J. Dutton J. Frost P. (2008). The contours and consequences of compassion at work. J. Organ. Behav. 29, 193–218. doi: 10.1002/job.508

[ref81] Luthans F. (2002). The need for and meaning of positive organizational behavior. J. Organ. Behav. 23, 695–706. doi: 10.1002/job.165

[ref82] Lyubomirsky S. King L. Diener E. (2005). The benefits of frequent positive affect: does happiness lead to success? Psychol. Bull. 131, 803–855. doi: 10.1037/0033-2909.131.6.803, PMID: 16351326

[ref83] Madrid H. Patterson M. Birdi K. Leiva P. Kausel E. (2014). The role of weekly high-activated positive mood, context, and personality in innovative work behavior: A multilevel and interactional model. J. Organ. Behav. 35, 234–256. doi: 10.1002/job.1867

[ref84] Maisel N. Gable S. (2009). “For richer … in good times … and in health: Positive processes in relationships,” in The Oxford Handbook of Positive Psychology. eds. Shane J. Snyder C. (New York: Oxford University Press), 455–462.

[ref85] Major B. Le Nguyen K. Lundberg K. Fredrickson B. (2018). Well-being correlates of perceived positivity resonance: evidence from trait and episode-level assessments. Personal. Soc. Psychol. Bull. 44, 1631–1647. doi: 10.1177/0146167218771324, PMID: 29756547 PMC8750237

[ref86] Mayer J. D. Salovey P. (1997). “What is emotional intelligence?” in Emotional Development and Emotional Intelligence: Educational Implications. eds. Salovey P. Sluyter D. J. (New York: Basic Books), 3–4.

[ref87] Mccullough M. Emmons R. Tsang J.-A. (2002). The grateful disposition: A conceptual and empirical topography. J. Pers. Soc. Psychol. 82, 112–127. doi: 10.1037/0022-3514.82.1.112, PMID: 11811629

[ref88] McPherson F. Janoff-Bulman R. (2000). Considering both sides: The limits of perspective taking. Basic Appl. Soc. Psychol. 22, 31–42. doi: 10.1207/S15324834BASP2201_4

[ref89] Miller L. Berg J. (1984). “Selectivity and urgency in interpersonal exchange,” in Communication, Intimacy, and Close Relationships. ed. Derlega V. J. (New York: Academic Press), 161–206.

[ref90] Molenberghs P. Bosworth R. Nott Z. Louis W. Smith J. Amiot C.. (2014). The influence of groups membership and individual differences in psychopathy and perspective taking on neural responses when punishing and rewarding others. Hum. Brain Mapp. 35, 4989–4999. doi: 10.1002/hbm.22527, PMID: 24753026 PMC6869684

[ref91] Monfort S. Kaczmarek L. Kashdan T. Drazkowski D. Kosakowski M. Guzik P.. (2014). Capitalizing on the success of romantic partners: A laboratory investigation on subjective, facial, and physiological emotional processing. Personal. Individ. Differ. 68, 149–153. doi: 10.1016/j.paid.2014.04.028

[ref92] Morelli S. Lieberman M. Zaki J. (2015). The emerging study of positive empathy. Soc. Personal. Psychol. Compass 9, 57–68. doi: 10.1111/spc3.12157

[ref93] Morelli S. Rameson L. Lieberman M. (2014). The neural components of empathy: predicting daily prosocial behavior. Soc. Cogn. Affect. Neurosci. 9, 39–47. doi: 10.1093/scan/nss088, PMID: 22887480 PMC3871722

[ref94] Murphy B. Costello T. Lilienfeld S. (2018). Is empathic contagion helpful or harmful? Overlooked heterogeneity in the empathy index. Psychol. Assess. 30, 1703–1708. doi: 10.1037/pas0000641, PMID: 30035555

[ref95] Neff K. Vonk R. (2009). Self-compassion versus global self-esteem: two different ways of relating to oneself. J. Pers. 77, 23–50. doi: 10.1111/j.1467-6494.2008.00537.x, PMID: 19076996

[ref96] O’Donohoe S. Turley D. (2006). Compassion at the counter: service providers and bereaved consumers. Hum. Relat. 59, 1429–1448. doi: 10.1177/0018726706071648

[ref97] O’Donovan R. McAuliffe E. (2020). A systematic review exploring the content and outcomes of interventions to improve psychological safety, speaking up and voice behavior. BMC Health Serv. Res. 20, 101. doi: 10.1186/s12913-020-4931-2, PMID: 32041595 PMC7011517

[ref98] Parker S. Axtell C. (2001). Seeing another viewpoint: antecedents and outcomes of employee perspective taking. Acad. Manag. J. 44, 1085–1100. doi: 10.2307/3069390

[ref99] Peters B. Reis H. Gable S. (2018). Making the good even better: A review and theoretical model of interpersonal capitalization. Soc. Personal. Psychol. Compass 12, 1–19. doi: 10.1111/spc3.12407

[ref100] Porath C. Spreitzer G. Gibson C. Garnett F. (2012). Thriving at work: Toward its measurement, construct validation, and theoretical refinement. J. Organ. Behav. 33, 250–275. doi: 10.1002/job.756

[ref101] Powley E. (2009). Reclaiming resilience and safety: resilience activation in the critical period of crisis. Hum. Relat. 62, 1289–1326. doi: 10.1177/0018726709334881

[ref102] Quinn R. Dutton J. (2005). Coordination as energy-in-conversation: A process theory of organizing. Acad. Manag. Rev. 30, 36–57. doi: 10.5465/amr.2005.15281422

[ref103] Ramarajan L. Barsade S. Burak O. (2008). The influence of organizational respect on emotional exhaustion in the human services. J. Posit. Psychol. 3, 4–18. doi: 10.1080/17439760701750980

[ref104] Rawls J. (1971). A Theory of Justice. Cambridge, MA: Belknap Press.

[ref105] Reis H. Shaver P. (1988). “Intimacy as an interpersonal process,” in Handbook of Personal Relationships: Theory, Research and Interventions. eds. Duck S. Hay D. Hobfoll S. Ickes W. Montgomery B. (Chichester: Wiley), 367–389.

[ref106] Reis H. Smith S. Carmichael C. Caprariello P. Tsai F.-F. Rodrigues A.. (2010). Are you happy for me? How sharing positive events with others provides personal and interpersonal benefits. J. Pers. Soc. Psychol. 99, 311–329. doi: 10.1037/a0018344, PMID: 20658846

[ref107] Rimé B. (2007). “Interpersonal emotion regulation,” in Handbook of Emotion Regulation. ed. Gross J. (New York: Guilford Press), 466–485.

[ref109] Rubin R. S. Munz D. C. Bommer W. H. (2005). Leading from within: The effects of emotion recognition and personality on transformational leadership behavior. Acad. Manag. J. 48, 845–858. doi: 10.5465/amj.2005.18803926

[ref110] Sallquist J. Eisenberg N. Spinrad T. Eggum N. Gaertner B. (2009). Assessment of preschoolers’ positive empathy: concurrent and longitudinal relations with positive emotion, social competence, and sympathy. J. Posit. Psychol. 4, 223–233. doi: 10.1080/17439760902819444, PMID: 20011674 PMC2790189

[ref111] Schaubroeck J. Lam S. (2004). Comparing lots before and after: promotion rejectees’ invidious reactions to promotes. Organ. Behav. Hum. Decis. Process. 94, 33–47. doi: 10.1016/j.obhdp.2004.01.001

[ref112] Sekerka L. Vacharkulksemsuk T. Fredrickson B. (2011). “Positive emotions: broadening and building upward spirals of sustainable enterprise,” in Handbook of Positive Organizational Scholarship. eds. Cameron K. Spreitzer G. (New York: Oxford University Press), 1–17.

[ref113] Sheppes G. Scheibe S. Suri G. Gross J. J. (2011). Emotion-regulation choice. Psychol. Sci. 22, 1391–1396. doi: 10.1177/0956797611418350, PMID: 21960251

[ref114] Singer T. Klimecki O. (2014). Empathy and compassion. Curr. Biol. 24, R875–R878. doi: 10.1016/j.cub.2014.06.054 25247366

[ref115] Stephens J. Heaphy E. Dutton J. (2011). “High quality connections,” in Handbook of Positive Organizational Scholarship. eds. Cameron K. Spreitzer G. (New York: Oxford University Press), 1–28.

[ref116] Tai K. Narayanan J. McAllister D. (2012). Envy as pain: rethinking the nature of envy and its implications for employees and organizations. Acad. Manag. Rev. 37, 107–129. doi: 10.5465/amr.2009.0484

[ref117] Tamir M. (2016). Why do people regulate their emotions? A taxonomy of motives in emotion regulation. Personal. Soc. Psychol. Rev. 20, 199–222. doi: 10.1177/1088868315586325, PMID: 26015392

[ref118] Taylor S. E. (1991). Asymmetrical effects of positive and negative events: The mobilization-minimization hypothesis. Psychol. Bull. 110, 67–85. doi: 10.1037/0033-2909.110.1.67, PMID: 1891519

[ref119] Telle N. Pfister H. (2016). Positive empathy and prosocial behavior: A neglected link. Emot. Rev. 8, 154–163. doi: 10.1177/1754073915586817

[ref120] Tesser A. (2000). On the confluence of self-esteem maintenance mechanisms. Personal. Soc. Psychol. Rev. 4, 290–299. doi: 10.1207/S15327957PSPR0404_1

[ref121] Turner J. (1975). Social comparison and social identity: Some prospects for intergroup behaviour. Eur. J. Soc. Psychol. 5, 1–34. doi: 10.1002/ejsp.2420050102

[ref122] Varnum M. Shi Z. Chen A. Qiu J. Han S. (2014). When “your” reward is the same as “my” reward: self-construal priming shifts neural responses to own vs. friends’ rewards. NeuroImage 87, 164–169. doi: 10.1016/j.neuroimage.2013.10.042, PMID: 24185022

[ref123] Waugh C. Fredrickson B. (2006). Nice to know you: positive emotions, self-other overlap, and complex understanding in the formation of new relationships. J. Posit. Psychol. 1, 93–106. doi: 10.1080/17439760500510569, PMID: 21691460 PMC3117671

[ref124] Weick K. (2012). Organized sensemaking: a commentary on processes of interpretive work. Hum. Relat. 65, 141–153. doi: 10.1177/0018726711424235

[ref125] Weick K. Sutcliffe K. Obstfeld D. (2005). Organizing and the process of sensemaking. Organ. Sci. 16, 409–421. doi: 10.1287/orsc.1050.0133

[ref126] Weiss H. Cropanzano R. (1996). Affective events theory: A theoretical discussion of the structure, causes and cosequences of affective experiences at work. Res. Organ. Behav. 18, 1–74.

[ref127] Worline M. Dutton J. (2017). Awakening Compassion at Work: The Quiet Power that Elevates People and Organizations. San Francisco, CA: Berrett-Koehler Publishers.

